# Supporting health promotion practitioners to undertake evaluation for program development

**DOI:** 10.1186/1471-2458-14-1315

**Published:** 2014-12-23

**Authors:** Roanna Lobo, Mark Petrich, Sharyn K Burns

**Affiliations:** WA Centre for Health Promotion Research, School of Public Health, Curtin University, GPO Box U1987, Bentley, WA 6845 Western Australia; Department of Health Policy and Management, School of Public Health, Curtin University, GPO Box U1987, Bentley, WA 6845 Western Australia

**Keywords:** Evaluation, Health promotion, Program development

## Abstract

**Background:**

The vital role of evaluation as integral to program planning and program development is well supported in the literature, yet we find little evidence of this in health promotion practice. Evaluation is often a requirement for organisations supported by public funds, and is duly undertaken, however the quality, comprehensiveness and use of evaluation findings are lacking. Practitioner peer-reviewed publications presenting evaluation work are also limited. There are few published examples where evaluation is conducted as part of a comprehensive program planning process or where evaluation findings are used for program development in order to improve health promotion practice.

**Discussion:**

For even the smallest of programs, there is a diverse array of evaluation that is possible before, during and after program implementation. Some types of evaluation are less prevalent than others. Data that are easy to collect or that are required for compliance purposes are common. Data related to how and why programs work which could be used to refine and improve programs are less commonly collected. This finding is evident despite numerous resources and frameworks for practitioners on how to conduct effective evaluation and increasing pressure from funders to provide evidence of program effectiveness. We identify several organisational, evaluation capacity and knowledge translation factors which contribute to the limited collection of some types of data. In addition, we offer strategies for improving health promotion program evaluation and we identify collaboration of a range of stakeholders as a critical enabler for improved program evaluation.

**Summary:**

Evaluation of health promotion programs does occur and resources for how to conduct evaluation are readily available to practitioners. For the purposes of program development, multi-level strategies involving multiple stakeholders are required to address the organisational, capacity and translational factors that affect practitioners’ ability to undertake adequate evaluation.

## Background

*“Evaluation’s most important purpose is not to prove but to improve”* ([1], p.4)*.*

The vital role of evaluation as integral to program planning and program development is well supported in the literature [2–5], yet we find little evidence of this in health promotion practice. Practitioners often cite a lack of time by staff already under significant work pressures as a reason why they do not conduct more evaluation [5]. Some practitioners also admit to undervaluing evaluation in the context of other competing priorities, notably service delivery. In our experience as evaluators, supported by the literature [6], we have found few examples where evaluation is part of a comprehensive program planning process or where program evaluation provides sufficient evidence to inform program development to improve health promotion practice.

**Figure 1:**
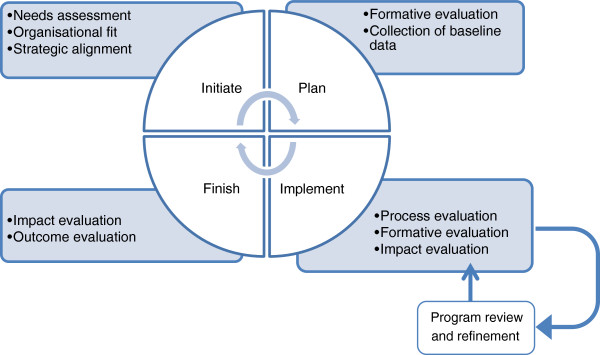
**Planning and evaluation cycle (adapted from** [8] **).**

There is a cyclical and ongoing relationship between program planning and evaluation. Different types of evaluation (formative, process, impact, outcome) are required at different stages in planning and implementing a program. Evaluation activities are much broader than measuring before and after changes as a result of intervention. They include stakeholder consultation, needs assessment, prototype development and testing, measuring outputs, monitoring implementation activities, and assessing how a program works, its strengths and limitations [7]. Following implementation, periods of reflection inform future program planning and refinement (see Figure 1, based on a four stage project management life cycle [8]).

This cyclical approach to planning and evaluation is not always evident in practice; many organisations appear to take a linear approach, often undertaking planning and evaluation activities independently, and commensurate with the funding available. Others see evaluation as a measure of compliance or proof that is more useful to those requesting or funding the program evaluation than to those implementing the program.

In this discussion we will argue that, for even the smallest of programs, there is a diverse array of evaluation that is possible before, during and after program implementation. We suggest that some types of evaluation are less prevalent than others. We will examine three types of factors contributing to limited program evaluation and propose strategies to support practitioners to undertake evaluation that improves health promotion practice.

## Discussion

Evaluation of health promotion programs does occur. However, the evaluation undertaken seems to be determined, at least in part, by data that is easy to collect, such as program costs, client satisfaction surveys, number of participants, number of education sessions, and costs per media target audience ratings points (TARPs). These data may be aggregated and reported on, however such cases seem to be fairly rare and restricted to evaluations that are undertaken as research or where compliance is required; for example, where program funding may not be approved without a clear evaluation plan.

There is little evidence of evaluation being truly used for program development despite:

*“Effective evaluation is not an ‘event’ that occurs at the end of a project, but is an ongoing process which helps decision makers better understand the project; how it is impacting participants, partner agencies and the community; and how it is being influenced/impacted by both internal and external factors”* ([26], p.3)*.*

- Extensive literature on the health promotion planning and evaluation process [7, 9–19];
- Evaluation identified as a core competency of health promotion practice [20–23]; and
- Planning and evaluation units as central components of public health and health promotion degrees [24, 25].

Our experience as evaluators indicates that practitioners frequently regard evaluation as an add-on process when a program is finished, rather than an ongoing process. Evaluation measures which are focused on outputs do not usually reflect the totality of a program’s effects. As such, practitioners may not perceive the data to be useful and therefore may undertake data collection solely to be compliant with program reporting requirements, or to justify continued funding.

Evaluation that identifies what works well and why, where program improvements are needed and whether a program is making a difference to a health issue will provide more valuable data for service providers. It is acknowledged that these data are often harder to collect than measures of program activity. However, these types of evaluation are needed to avoid missed opportunities for service improvements [6]. Important evaluation questions include: *Are resources being used well? Are services equitable and accessible? Are services acceptable to culturally and linguistically diverse populations? Could modifications to the program result in greater health gains?*

The answers to these types of questions can inform more effective and sustainable health promotion programs, reduce the potential for iatrogenic effects (preventable harm), and justify the expenditure of public funds. To be an ethical and responsible service provider, this accountability is critical.

*Why does the planning and evaluation cycle break down for some organisations?*

**Figure 2:**
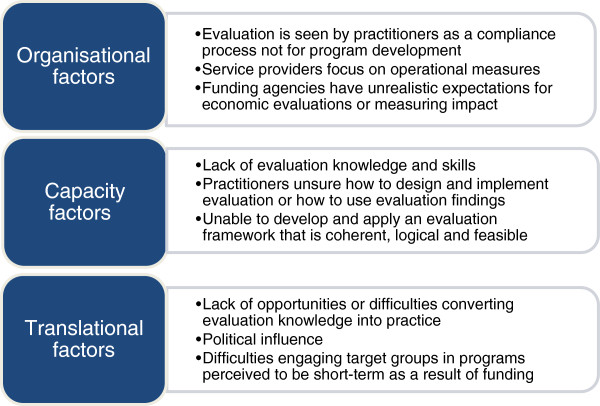
**Factors contributing to limited program evaluation.**

We can identify a number of factors that contribute to limited program evaluation, many of which are generally well known to health promotion practitioners [5, 6, 18, 19, 27, 28]. We have grouped these constraints on program evaluation broadly as organisational, capacity, and translational factors (see Figure 2).

### Organisational factors

Funding for preventive health programs is highly competitive [15], with implied pressure to pack in a great deal of intervention, often to the detriment of the evaluation and the health programs. Economic evaluations are also desired by funding agencies without clear appreciation of the technical constraints and costs involved in undertaking these evaluations [16, 25]. Since health promotion programs often do not attract significant funding or resources [23, 29], the evaluation budget can be limited. In addition, funding can operate on short-term cycles, often up to 12 months, see for example projects funded by the Western Australian Health Promotion Foundation (Healthway) [30].

These project-based funding models can damage the sustainability of good preventive health programs for a number of reasons, including that funding may end before the program is able to demonstrate evidence that it works. Instead of desirable longer-term emphasis on health change and program development, evaluation choices tend to be responsive to these short-term drivers. Consequently, the planning process for programs may be fast-tracked with the focus weighted heavily towards intervention, and the evaluation component can move towards one of two extremes – either research-oriented or managerial evaluations.

Research-oriented evaluation projects, experimental in nature, include attempts to create a controlled research study, which may not be sustainable outside the funding period. There is good evidence of sound research-oriented evaluations [31, 32]. However, the gold standard of the randomised controlled trial (RCT) requiring a comparison non-intervention control group is not always appropriate for a health promotion program that is delivering services to everyone in the community [33]. Many health promotion programs are also not well suited to quasi-experimental evaluation of health outcomes, given their naturalistic setting and proposed program benefits often well into the future. The rigorous study design of research-oriented projects is important but may not be appropriate for many community and national health promotion interventions.

Managerial evaluations are minimalist types of evaluation that are designed to meet auditing requirements. We suspect that understanding of program evaluation has become rather superficial, based more on recipe-style classification or naming conventions (formative, process, impact, outcome evaluation) and less on the underlying purpose.

These categories may meet funder requirements as a recognisable model of types of evaluation. However, the purpose of the evaluation and its value are often lost. The capacity to inform both the current program and future iterations is also reduced. Furthermore, the usefulness of the evaluation data collected may be compromised if the program planning process has not considered evaluation in advance, in particular, objectives that are relevant to the project and that can be evaluated.

### Capacity factors

Evaluation of health promotion programs is particularly important to prevent unintended effects. Yet the reality for agencies undertaking health promotion work is that evaluation may not be well understood by staff, even those with a health promotion or public health background. For example, health promotion practitioners intending to collect evaluation data that can be shared, disseminated or reported more widely may need to partner with a relevant organisation in order to access a Human Research Ethics Committee (HREC) to seek ethics approval. Evaluation can also be seen as difficult [28, 34], and a task that cannot be done without specialist software, skills or assistance.

As a result, there may be inadequate consideration of evaluation or evaluation is not planned at all. Data are not always suitable to address desired evaluation questions, or are not collected well. Consider the use of surveys to obtain evaluation data. People creating surveys often have no training in good survey design. The resulting survey data are not always useful or very meaningful and these negative experiences may fuel service providers’ doubts about the value of evaluation surveys, and indeed evaluation in general.

A number of circumstances can contribute further to limited evaluation. For example, when resources are scarce or there is instability in the workforce, or when a program receives recurrent funding with no requirement for evidence of program effectiveness, the perceived value of rigorous evaluation is low. Furthermore, in any funding round, the program may no longer be supported making any evaluative effort seem wasteful. Conversely, if the program was successful in previous funding rounds there is often little motivation to change other than a gradual creep in activity number and/or scope. Consequently, there appear to be few incentives for quality evaluation used to inform program improvements, to modify, expand or contract, or to change direction in programming.

### Translational factors

Political influence also plays a role. Being seen to be ‘doing something quickly’ to address a public health issue is a common reaction by politicians leading to hurriedly planned programs with few opportunities for evaluation. The difficulties of working with hard to reach and unengaged target groups may be ignored in the urgency to act quickly. As a result, getting buy-in from community groups may not be actively sought, or may be tokenistic at best, with the potential for exacerbating the social exclusion experienced by marginalised groups [35].

There is also some reluctance by practitioners to evaluate due to potential risk of unfavourable findings. Yet, it is important to share both positive and negative outcomes so that others learn from and do not make similar mistakes. Confidence to publish lessons learned firstly requires developing the skills to be able to disseminate knowledge through peer-reviewed journals and other forums. Secondly, practitioners require reassurance from funding agencies that funds will not automatically be withdrawn without opportunity for practitioners to adjust programs to improve outcomes.

Information about how to conduct evaluations for different types of programs is plentiful and readily available to practitioners in the public domain (see for example [7, 9, 10, 16]). Strategies for building evaluation capacity and creating sustainable evaluation practice are also well documented [36]. Yet barriers to translating this knowledge into health promotion practice clearly remain [34, 37].

*What is needed to support practitioners to undertake improved evaluation?*

**Figure 3:**
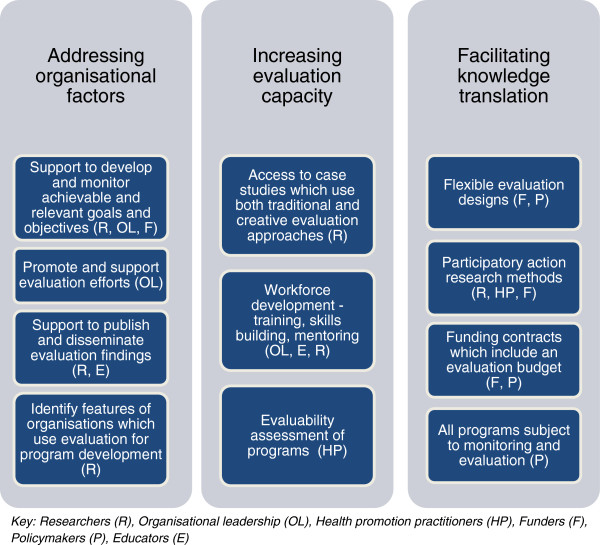
**Improving health promotion program evaluation.**

We propose that multi-level strategies are needed to address the organisational, capacity and translational factors that contribute to the currently limited program evaluation focused on health promotion program development [6, 12, 14]. We also suggest that supporting health promotion practitioners to conduct evaluations that are more meaningful for program development is a shared responsibility. We identify strategies and roles for an array of actors including health promotion practitioners, educators, policymakers and funders, organisational leadership and researchers. Many strategies also require collaboration between different roles. Examples of the strategies and shared responsibilities needed to improve health promotion program evaluation are shown in Figure 3 and are discussed below.

### Strategies to address organisational factors

We have questioned here the expectations of funding agencies in relation to evaluation. We concur with Smith [6] and question the validity of the outcomes some organisations may require, or expect, from their own programs. Assisting organisations to develop achievable and relevant goals and objectives, and processes for monitoring these, should be a focus of capacity building initiatives.

Organisational leadership needs to place a high value on evaluation as a necessary tool for continuous program development and improvement. There should be greater focus on quantifying the extent of impact needed. Practitioners need to feel safe to ask: *could we be doing better?* Asking this question in the context of raising the standards and quality of programs to benefit the target groups [6] is recommended rather than a paradigm of individual or group performance management.

Establishing partnerships with researchers can add the necessary rigour and credibility to practitioners’ evaluation efforts and can facilitate dissemination of results in peer-reviewed journal articles and through conferences. Sharing both the processes and results of program evaluations in this way is especially important for the wider health practitioner community. Furthermore, identifying organisations that use evaluation well for program development may assist in understanding the features of organisations and the strategies and practices needed to overcome common barriers to evaluation.

### Strategies to increase evaluation capacity

In some organisations, there is limited awareness of what constitutes evaluation beyond a survey, or collecting operational data. We would argue that with some modification, many existing program activities could be used for evaluation purposes to ensure systematic and rigorous collection of data. Examples include recording journal entries of program observations, and audio or video recording of data to better understand program processes and participant involvement.

Specialist evaluation skills are not always required. Practitioners may wish to consider appreciative inquiry methods [38] which focus on the strengths of a program and what is working well rather than program deficits and problems. Examples include the *most significant change* technique, a highly participatory story-based method for evaluating complex interventions [39] and the *success case method* for evaluating investments in workforce training [40]. These methods use storytelling and narratives and provide powerful participatory evaluation methods for integrating evaluation and program development. Arts-based qualitative inquiry methods such as video diaries, graffiti/murals, theatre, photography, dance and music are increasingly being explored for their use in health promotion given the relationship between arts engagement and health outcomes [41]. Boydell and colleagues provide a useful scoping review of arts-based health research [42].

Such arts-based evaluation strategies may be particularly suited to programs that already include creative components. They may also be more culturally acceptable when used as community engagement tools for groups where English is not the native language or where literacy may be low. In our experience, funding agencies are increasingly open to the validity of these data and their potential for wider reach, particularly in vulnerable populations [43, 44]. The outputs of arts-based methods (for example, photography exhibitions, theatre performances) are also powerful channels for disseminating results and have the potential to influence policy if accepted as rigorous forms of evidence [42].

We encourage practitioners to begin a dialogue with funders to identify relevant methods of evaluation and types of evidence that reflect what their programs are actually doing and that provide meaningful data for both reporting and program development purposes. Other authors have also recognised the paucity of useful evaluations and have developed a framework for negotiating meaningful evaluation in non-profit organisations [45].

Workforce development strategies, including mentoring, training, and skills building programs, can assist in capacity building. There are several examples of centrally coordinated capacity building projects in Australia which aim to improve the quality of program planning and evaluation in different sectors through partnerships and collaborations between researchers, practitioners, funders and policymakers (see for example SiREN, the Sexual Health and Blood-borne Virus Applied Research and Evaluation Network [46, 47]; the Youth Educating Peers project [48]; and the REACH partnership: Reinvigorating Evidence for Action and Capacity in Community HIV programs [49]). These initiatives seek to provide health promotion program planning and evaluation education, skills and resources, and assist practitioners to apply new knowledge and skills. The Western Australian Centre for Health Promotion Research (WACHPR) is also engaged in several university-industry partnership models across a range of sectors including injury prevention, infectious diseases, and Aboriginal maternal and child health.

These models have established formal opportunities for health promotion practitioners to work alongside health promotion researchers, for example, co-locating researchers and practitioners to work together over an extended period of time. Immersion and sharing knowledge in this way seeks to enhance evaluation capacity and evidence-based practice, facilitate practitioner contributions to the scholarly literature, and improve the relevance of research for practice. There has been some limited evaluation of these capacity building initiatives and it is now timely to collect further evidence of their potential value to justify continued investment in these types of workforce development strategies.

Also important is evaluability assessment, including establishing program readiness for evaluation [7] and the feasibility and value of any evaluation [22]. Though rare, in some cases, agencies may over-evaluate without putting the results to good use. Organisations need to be able to assess when to evaluate, consider why they are evaluating, and be mindful whether evaluation is needed at all. Clear evaluation questions should always guide data collection. The use of existing data collection tools which have been proven to be reliable is advantageous for comparative purposes [7].

### Strategies to facilitate knowledge translation

Evaluation has to be timely and meaningful, not simply confirming what practitioners know, otherwise there is limited perceived value in conducting evaluation. Practical evaluation methods that can be integrated into daily activities work well and may be more sustainable by practitioners [50].

It is not always possible to collect baseline data. Where no baseline data has been collected against which to compare results, this does not have to be a barrier to evaluation, as comparisons against local and national data may be possible. Post-test only data, if constructed well, can also provide some indication of effectiveness, for example, collecting data on ratings of improved self-efficacy as a result of a project.

Practitioners should be encouraged that evaluation is a dynamic and evolving process. Evaluation strategies may need to be adapted several times before valuable data are collected. This process of reflective practice may be formalised in the approach of participatory action research [51] which features cycles of planning, implementation and reflection to develop solutions to practical problems. Through active participation and collaboration with researchers, practitioners build evaluation skills and increased confidence that evaluation is within their capability and worth doing to improve program outcomes.

Funding rules can help reinforce the importance of adequate evaluation as an essential component of program planning. Some grants require 10-15% of the program budget to be spent on evaluation including translation of evaluation findings. Healthway, for example, encourages grant applicants to seek evaluation planning support from university-based evaluation consultants prior to submitting a project funding application.

The Ottawa Charter for Health Promotion guides health promotion practice to consider multi-level strategies at individual, community and system levels to address complex issues and achieve sustainable change [52]. The role of political influence on the strategies that are implemented and the opportunities for evaluation cannot, however, be ignored. A balance must be achieved between responding quickly to a health issue attracting public concern and undertaking sufficient planning to ensure the response is appropriate and sustainable. In a consensus study with the aim of defining sustainable community-based health promotion practice, practitioners highlighted four key features: effective relationships and partnerships; building community capacity; evidence-based decision-making and practice; and a supportive context for practice [29]. Reactive responses may be justified if implemented as part of a more comprehensive, evidence-based and theory-based health promotion strategy. A significant evaluation component to assess the responses can enable action to extend, modify or withdraw the strategies as appropriate.

## Summary

In this article, we have argued that along the spectrum of evaluation, evaluation activities focused on output measures are more frequently undertaken than evaluation activities that are used to inform program development. We have outlined organisational, capacity, and translational factors that currently limit the extent of program evaluation undertaken by health promotion practitioners.

We propose that multiple strategies are needed to address the evaluation challenges faced by health promotion practitioners and that there is a shared responsibility of a range of stakeholders for building evaluation capacity in the health promotion workforce (Figure 3). We conclude that adequate evaluation resources are available to practitioners; what is lacking is support to apply this evaluation knowledge to practice.

The types of support needed can be identified at an individual, organisational, and policy level:

- Individual practitioners require support to develop evaluation knowledge and skills through training, mentoring and workforce capacity development initiatives. They also need organisational leadership that endorses evaluation activities as valuable (and essential) for program development and not conducted simply to meet operational auditing requirements.
- Organisations need to create a learning environment that encourages and rewards reflective practice and evaluation for continuous program improvement. In addition, a significant evaluation component is required to ensure that reactive responses to major public health issues can be monitored, evaluated and modified as required.
- At a policy level, all programs should be monitored, evaluated and refined or discontinued if they do not contribute to intended outcomes. Seeking evidence that is appropriate to program type and stage of development is important; funders need to give consideration to a range of evaluation data. Adequate budget also needs to be available for and invested in program evaluation.

Evaluation of health promotion programs does occur and resources for how to conduct evaluations are readily available to practitioners. Multi-level strategies involving a range of stakeholders are also required to address the organisational, capacity and translational factors that influence practitioners’ ability to undertake adequate evaluation. Establishing strong partnerships between key stakeholders who build evaluation capacity, fund or conduct evaluation activities will be critical to support health promotion practitioners undertake evaluation for program development.

## Abbreviations

HREC: Human Research Ethics Committee
RCT: Randomised Controlled Trial
REACH: Reinvigorating Evidence for Action and Capacity in Community HIV programs
SiREN: Sexual Health and Blood-borne Virus Applied Research and Evaluation Network
TARPs: Target audience ratings points
WACHPR: Western Australian Centre for Health Promotion Research
